# One-year safety and quality of life outcomes in patients with atrial fibrillation on dronedarone: prospective, non-interventional study in German ambulatory care

**DOI:** 10.1007/s00399-015-0360-z

**Published:** 2015-03-08

**Authors:** Andreas Goette, G. Benninger, D. Pittrow, W.D. Paar, B. von Stritzky, R.F. Bosch

**Affiliations:** 1 Department of Cardiology and Intensive Care Medicine, Medizinische Klinik II, St. Vincenz-Hospital Paderborn GmbH, Am Busdorf 2, 33098 Paderborn, Germany; 2 Atrial Fibrillation Competence Network (AFNET) Association, Münster, Germany; 3 Institute for Clinical Pharmacology, Medical Faculty, Technical University Carl Gustav Carus, Dresden, Germany; 4 Medical Department, Sanofi-Aventis Deutschland GmbH, Berlin, Germany; 5 Cardio Centrum Ludwigsburg-Bietigheim, Ludwigsburg, Germany

**Keywords:** Atrial fibrillation, Treatment, Observational, Quality of life, Long term, Safety, Outcomes, Vorhofflimmern, Therapie, Beobachtungsstudie, Sicherheit, Lebensqualität, Langzeitergebnisse

## Abstract

**Background and aims:**

The multichannel blocker dronedarone is currently indicated for the maintenance of sinus rhythm after successful cardioversion in adult clinically stable patients with paroxysmal or persistent atrial fibrillation (AF), with careful monitoring of cardiac, hepatic and renal function. We aimed to investigate patients’ quality of life (QoL) and tolerability and effectiveness of dronedarone under real life conditions.

**Methods:**

In the 1-year prospective, non-interventional IMPULS study, 161 office-based cardiologists, general practitioners and internists throughout Germany documented 549 patients with AF who were currently or newly prescribed dronedarone (safety set, SS). Of those, 342 patients (full analysis set, FAS) provided data on QoL at baseline, 6 months and 12 months).

**Results:**

Mean age of patients was 67.6/66.3 years; 53.0 %/57.3 % were men (SS/FAS). AF type at inclusion in the SS/FAS was paroxysmal in 71.9 %/71.3 % and persistent in 26.0 %/26.6 % (missing in 2.0 %/2.0 %). The proportion of patients in sinus rhythm increased from 44.6 % at baseline to 70.2 % (SS). The mean value on the 100-point visual analogue scale (EuroQol EQ-5D) increased from 62.3 ± 17.1 at baseline by 11.4 ± 18.7 points (FAS, *p*<0.0001). The AF-QoL Psychological Domain improved from 44.6 ± 22.6 at baseline by 16.0 ± 23.5 points at 1 year (*p*<0.0001), the AF-QoL physical domain from 49.5 ± 22.1 by 10.9 ± 22.5 points (*p*<0.0001), and the AF-QoL sexual domain from 61.8 ± 27.1 by 6.6 ± 28.2 points (*p*<0.0001). In all, 136 patients (24.8 % of all patients in the safety set) had at least one adverse drug reaction (ADR) causally related to dronedarone.

**Conclusions:**

Various dimensions of quality of life of patients with AF were improved on dronedarone under clinical practice conditions. No previously unknown safety issues were noted.

## Abstract (de)

**Hintergrund und Ziele:**

Der Mehrkanal-blocker Dronedaron ist – nachdem alternative Behandlungsoptionen in Erwägung gezogen wurden – angezeigt zum Erhalt des Sinusrhythmus nach einer erfolgreichen Kardioversion bei erwachsenen, klinisch stabilen Patienten mit paroxysmalem oder persistierendem Vorhofflimmern (VHF). Die kardiale, hepatische und renale Funktion sind zu kontrollieren. Wir untersuchten die Lebensqualität der Patienten unter Dronedaron sowie die Verträglichkeit und Wirksamkeit der des Antiarrhythmikums unter Praxisbedingungen.

**Methoden:**

In der prospektiven, nicht-interventionellen IMPULS-Studie dokumentierten 161 niedergelassene Kardiologen, hausärztlich tätige Internisten und Allgemeinärzte in Deutschland 549 Patienten mit VHF (Safety Set (SS)), die bereits Dronedaron erhielten oder denen die Substanz erstmalig verschrieben wurde. Davon hatten 342 Patienten (Full-Analysis Set, FAS) Daten zur Lebensqualität zu Beginn der Studie sowie nach 6 und 12 Monaten.

Das mittlere Alter der Patienten lag bei 67,6/66,3 Jahren und 53,0 %/57,3 % waren Männer (SS/FAS). Hinsichtlich des VHF-Typs wurde die paroxysmale Manifestation bei 71,9 %/71,3 % und die persistierende bei 26,0 %/26,6 % angegeben (fehlende Angabe bei 2,0 %/2,0 %;) (SS/FAS). Der Anteil der Patienten im Sinusrhythmus nahm von 44,6 % bei Dokumentationsbeginn auf 70,2 % nach 1 Jahr zu (SS). Die Lebensqualität verbesserte sich auf der 100-stufigen visuellen Analogskala (EuroQol EQ-5D) von 62,3 ± 17,1 Punkten um 11,4 ± 18,7 Punkte (FAS; *p*<0,0001). Hinsichtlich der Dimension „psychische Beeinträchtigung“ im AF-QoL (Ausgangsmittelwert ± SD 44,6 ± 22,6) wurde eine Verbesserung von 16,0 ± 23,5 Punkten nach 1 Jahr dokumentiert. Bei „körperliche Beeinträchtigung“ (von 49,5 ± 22,2) betrug die Verbesserung 10,9 ± 22,5 Punkte (*p*<0.0001), und bei „Sexualität“ (von 61,8 ± 27,1 Punkten) 6,6 ± 28,2 Punkte (*p*<0.0001). Nebenwirkungen, die vom dokumentierenden Arzt als möglicherweise oder sicher in Beziehung zu Dronedaron stehend eingestuft wurden (unerwünschte Arzneimittelwirkungen, UAW), traten bei 136 Patienten auf (24,8 % des SS).

**Schlussfolgerung:**

Unter Alltagsbedingungen verbesserten sich verschiedene Dimensionen der Lebensqualität während der Therapie mit Dronedaron. Das Medikament wurde gut vertragen.

## Background

Atrial fibrillation (AF) is the most common sustained cardiac arrhythmia occurring in about 1 % of the general population (10 % of all people aged > 80 years are suffering from AF) [1]. Over 6 million Europeans suffer from this arrhythmia and its prevalence is estimated to increase significantly within the next decades as the population ages and manifests more comorbidities [2, 3].

AF is associated with a doubling of overall mortality and a fivefold increased risk of stroke [4, 5]. Clinical symptoms may include palpitations, dyspnoea or syncopes with significant impairment of quality of life (QoL). On the other hand AF can also occur unnoticed unless incidentally found or until complications occur [2, 6]. A number of newer investigations, which used a non-interventional design similar to ours, showed substantially compromised QoL in AF patients [7−12].

Dronedarone is an antiarrhythmic drug which has a benzofuran moiety as amiodarone but does not possess the iodine part affecting thyroid function [13]. Due to differences such as a methyl sulphonyl group the lipophilicity of the new agent compared with amiodarone was reduced and its plasma half-life substantially shortened thought to reduce organ toxicity due to cumulative effects.

The drug has been launched in 2010 in Germany under the brand name Multaq®. Based on the results of new trials and pharmacovigilance reports its labelling has been amended several times. According to the current summary of product characteristics, Multaq® is indicated for the maintenance of sinus rhythm after successful cardioversion in adult clinically stable patients with paroxysmal or persistent AF [14]. Due to its safety profile, the agent should only be prescribed after alternative treatment options have been considered. Multaq® should not be given to patients with left ventricular systolic dysfunction or to patients with current or previous episodes of heart failure [14]. Careful monitoring during dronedarone administration is recommended with regular assessment of cardiac, hepatic and pulmonary function.

While dronedarone has been extensively documented in the context of clinical studies [15], there is a paucity of data on the use under real life conditions, with the exception of a retrospective database analysis of all patients treated with the drug between 2010 and 2012 in Sweden [16]. The real life patient population often differs from patient cohorts in controlled clinical studies with regards to demographic characteristics, comorbidities and concomitant diseases. Data collected in non-interventional studies like IMPULS can complement the findings of pivotal studies. We aimed to collect such data, with particular focus on QoL.

## Methods

### Study design and timelines

IMPULS was a prospective multicentre non-interventional study (NIS) according to § 67 (6) German Drug Law (AMG) to document the management/treatment of consecutive patients treated with Multaq® over a period of 12 months. All procedures followed were in accordance with the ethical standards with the Helsinki Declaration of 1975 (in its most recently amended version). The study materials were reviewed and approved by the ethics committees of the Ärztekammer Westfalen-Lippe and of the Medical Faculty of Wilhelms University of Münster. Informed consent was obtained from all patients included in the study. The study was performed between January 2012 (first patient in) and December 2013 (last patient out).

### Centres

Office-based cardiologists, general practitioners and internists were eligible for participation. Selection of centres aimed to obtain a representative distribution with respect to geography and physician specialisation, respectively.

### Patients

Patients were eligible for documentation, if they (1) were newly treated with dronedarone or were on maintenance treatment with dronedarone no longer than 3 months; (2) had paroxysmal or persistent AF and at least one cardiovascular risk factor (arterial hypertension, diabetes mellitus, previous stroke, transient ischaemic attack, arterial embolism, left atrial diameter ≥ 50 mm); (3) had provided written informed consent to participate in the study. No explicit exclusion criteria were applied to avoid selection bias. All diagnoses were provided by the treating physician and were not adjudicated by third parties.

No diagnostic measures or treatment methods were stipulated, but remained in the sole responsibility of the participating physicians. At baseline and after approximately 6 and 12 months, respectively, the physicians documented diagnostic and therapeutic parameters as assessed under routine treatment or as available from additional sources such as, e.g., hospital reports or patient charts.

In addition, patients were asked to complete QoL questionnaires at baseline and at their 6-month (FU1) and 12-month follow-up (FU2) visits at the physician’s office. As generic instruments, the 100-point EQ-5D visual analogue scale (VAS) [17] and the short form 12 (SF-12) [18] were applied. Further, the AF-QoL questionnaire as disease-specific questionnaire was administered [19, 20].

### Parameters

At baseline, characteristics on demographics (gender, age and employment status), basic data and vital signs, cardiac risk factors, cardiac history and concomitant diseases were noted. Particular focus was on hepatic function (recording of last alanine aminotransferase (ALT) values, renal function (creatinine value)), and in the case of vitamin K treatment, the international normalised ratio.

With regards to AF, the following information was documented: month of first diagnosis, type, current rhythm according to last ECG and symptoms. Further, therapy within the last 12 months, hospitalisations due to AF or other reasons, current therapy for the prevention of thromboembolic complications, management of AF in the last 12 months were documented. At 3 months, ALT and creatinine values between the initiation of dronedarone therapy and the visit were noted.

At the two FU visits at 6 months and 12 months, physicians predominantly documented current symptoms, therapy for AF and anticoagulation, newly occurring vascular events (transient ischaemic attacks, stroke, myocardial infarction, heart failure or other cardiac events) and detailed information on cardioversion or other therapy. Medication for rhythm control of AF was recorded by Vaughan-Williams classification.

### Data entry and analysis

Data were collected using paper–pencil case record forms. Duplicate data entry was performed by the contract research organisation, and plausibility checks were executed using a validation plan.

**Fig. 1:**
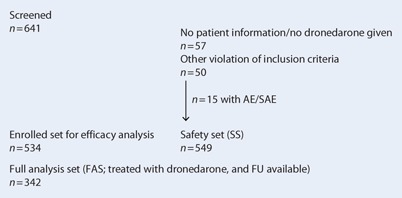
Patient disposition. *AE* adverse event, *SAE* serious adverse event, *FU* follow-up

The safety set consisted of 549 patients, the enrolled set of 534 patients and the full analysis set of all patients treated at least once with dronedarone and FU data available for 342 patients (Fig. 1).

### Statistical analysis

Continuous variables are reported as mean with standard deviation, categorical variables as percentage of patient population. Due to incomplete answers and multiple answering options, observed numbers and percentages do not always add up to exactly 100 %. Comparisons between baseline and FU were performed with the two-tailed Student’s paired sample *t*-test. Data were analysed using the SAS statistical package Version 9.2.

## Results

A total of 161 office-based cardiologists, general practitioners and internists throughout Germany took part in this study.

### Patient characteristics

Characteristics of patients are displayed in Table 1. Mean age of patients was 66.3 years, 57.3 % were men. Patients had paroxysmal AF in 71.4 % and persistent AF in 26.6 % (missing 2.1 %). Comorbidities were frequent, in particular arterial hypertension, coronary artery disease and diabetes mellitus.

**Table 1:** Demographic variables AF type and comorbidies inclusion (FAS, *n* = 342)

Variable	*n*	Value
Age, years (mean)	342	66.3 ± 9.7
< 65 years (%)	134	39.2
≥ 65 years (%)	208	60.8
Gender, male (%)	196	57.3
Female (%)	146	42.7
Body mass index, kg/m^2^ (mean ± SD)	341	28.4 ± 4.3
**AF type (%)**		
Paroxysmal	244	71.4
Persistent	91	26.6
Missing	7	2.1
AF duration, days (median)	334	396
**Comorbidities (%)**		
Arterial hypertension	321	93.9
Diabetes mellitus	80	23.4
Hyperthyreosis	13	3.8
Pathological alcohol consumption	4	1.2
Stroke/TIA	24	7.0
Left atrial diameter ≥ 50 mm	47	13.7
Valvular defect	60	17.5
Coronary artery disease	74	21.6

### Primary effectiveness variables (FAS)

#### AF-QoL

The AF-QoL psychological domain improved from 44.6 ± 22.6 at baseline to 56.7 ± 21.7 at FU1 (i.e. + 12.1 ± 20.8 points, *p*<0.0001), and to 60.6 ± 22.6 at FU2 (+ 16.0 ± 23.5 points, *p*<0.0001). Further, the AF-QoL physical domain improved from 49.5 ± 22.2 at baseline to 59.8 ± 20.5 at FU1 (i.e. + 10.3 ± 19.5 points, *p*<0.0001), and to 60.3 ± 24.0 at FU2 (+ 10.9 ± 22.5 points, *p*<0.0001). Finally, the AF-QoL sexual domain improved from 61.8 ± 27.1 at baseline to 68.3 ± 24.8 at FU1 (i.e. + 6.5 ± 24.2, *p*<0.0001), and to 68.4 ± 26.7 at FU2 (+ 6.6 ± 28.2 points, *p*<0.0001).

#### EQ-5D

The mean value on the 100-point VAS increased from 62.3 ± 17.1 at baseline to 73.1 ± 17.0 at FU1 (+ 10.8 ± 17.9 points, *p*<0.0001) and to 73.9 ± 17.3 at FU2 (+ 11.4 ± 18.7 points, *p*<0.0001).

For both the AF-QoL and the EQ-5D, there were no important differences between men and women or in patients with paroxysmal versus persistent AF.

### Secondary effectiveness variables

#### SF-12

The mean SF-12 physical summary scale increased from 42.3 ± 8.6 points at baseline to 46.2 ± 7.9 at FU1 (+ 4.0 ± 8.6 points, *p*<0.0001) and to 46.5 ± 9.0 (+ 4.3 ± 9.3 points, *p*<0.0001) at FU2. The mental summary scale increased from 43.4 ± 11.9 points at baseline to 47.9 ± 10.0 at FU1 (+ 4.4 ± 10.3 points, *p*<0.0001) and to 48.1 ± 9.8 (+ 4.8 ± 11.3 points, *p*<0.0001) at FU2.

#### Rhythm control rates

The proportion of patients in sinus rhythm increased from 44.6 % at baseline to 70.2 % at FU1 and 70.9 % at FU2.

#### General health evaluation

**Fig. 2:**
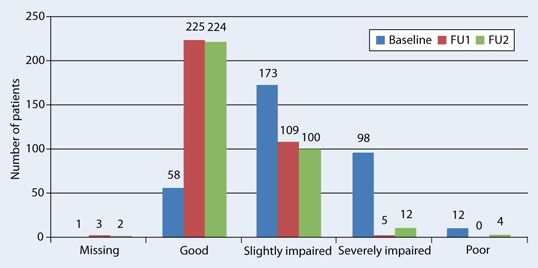
Self-reported health state of patients at baseline, at follow-up 1 (6 months) and follow-up 2 (12 months)

While at baseline, the great majority of patients reported at least slight or moderate impairment of their general health, at FU their self-reported state had considerably improved (Fig. 2).

### Safety and tolerability (safety set)

#### Overall assessment

A total of 281 individual adverse drug reactions causally related to Multaq®, of which 165 were rated as serious and 116 as non-serious, were reported in 136 patients (24.6% of all patients in the safety set, Table 2). Most serious ADRs (SADR) occurred in the category ‘cardiac disorders’ (94 SADRs), followed by ‘general disorders and administration site conditions’ (23 SADRs), ‘investigations’ (18 SADRs) and ‘respiratory, thoracic and mediastinal disorders’ (11 SADRs).

AF was the most frequently noted SADR (in 13.8 % of patients). All other events occurred in less than 2 % (e.g. drug ineffectiveness in 1.5 %, heart failure in 1.3 % and dyspnoea in 1.3 %). The most frequent non-serious ADRs were increased ALT (2.0 % of patients), nausea (1.3 %) and diarrhoea (1.3 %); all other events were noted less than 1 %.

Out of the 136 patients with SADR or ADR, a total of 110 discontinued Multaq® therapy. The most frequently noted underlying conditions (Table 3) were AF (10.4 % of patients), increased ALT (2.2 % of patients), dyspnoea (2.2 % of patients) and drug ineffectiveness (1.6 % of patients).

**Table 2:** Overview on adverse drug reactions (ADR)

Category	Events (*n*)	Patients (*n*)	% of patients at risk
**Any ADR related**	281	136	24.8
**Non-serious ADR**	116	57	10.4
**Serious ADR**	165	92	16.8

**Table 3:** ADR leading to withdrawal of Multaq® (safety set)

System Organ Class	Term	Frequency	% of patients
Cardiac disorders		80	
	Arrhythmia	2	0.36
	Atrial fibrillation	60	10.38
	Bradycardia	2	0.36
	Cardiac failure	7	1.28
	Left ventricular dysfunction	2	0.36
	Palpitations	3	0.55
	Tachyarrhythmia	2	0.36
General disorders and administration site conditions		39	
	Condition aggravated	2	0.36
	Drug ineffective	10	1.64
	Drug intolerance	4	0.73
	Fatigue	2	0.36
	General physical health deterioration	2	0.36
	Ill-defined disorder	4	0.73
	Local swelling	2	0.36
	Malaise	3	0.55
	Oedema peripheral	2	0.36
Investigations		37	
	Alanine aminotransferase ↑	12	2.19
	Aspartate aminotransferase ↑	2	0.36
	Blood creatinine ↑	3	0.55
	Gamma-glutamyltransferase ↑	2	0.36
	Hepatic enzyme ↑	4	0.73
	International normalised ratio ↑	2	0.36
	Liver function test abnormal	3	0.55
	Transaminases ↑	4	0.73
Gastrointestinal disorders		27	
	Abdominal discomfort	5	0.91
	Abdominal pain upper	2	0.36
	Diarrhoea	7	1.28
	Gastrointestinal disorder	2	0.36
	Nausea	7	1.28
Respiratory, thoracic and mediastinal disorders		17	
	Dyspnoea	12	2.19
	Dyspnoea exertional	2	0.36
	Interstitial lung disease	2	0.36
Nervous system disorders		11	
	Dizziness	4	0.73
	Headache	2	0.36
	Syncope	3	0.55
Skin and subcutaneous tissue disorders		11	
	Hyperhidrosis	2	0.36
	Rash	3	0.55

Table shows reactions that occurred in more than 1 patient. ↑, increased

#### Laboratory values

ALT values were increased at least 3 times above the upper reference limit in 60 patients (10.9 %). There was no specific time pattern in the occurrence of the elevations.

Elevations of creatinine at least 2 times above the upper reference limit were not documented in this study.

## Discussion

To the best of our knowledge, the IMPULS study is the only prospective observational study that specifically documents the use of dronedarone under clinical practice conditions. Previous similar AF studies in Germany such as MOVE [21] or ATRIUM [22] were performed too early to accrue significant patient numbers.

IMPULS used similar inclusion criteria as the ATHENA study and focused on patients with AF who had additional risk factors for death [23]. In that study, patients in the dronedarone group had higher rates of bradycardia, QT-interval prolongation, nausea, diarrhoea, rash and an increased serum creatinine level than the placebo group, whereas rates of thyroid- and pulmonary-related adverse events were not significantly different between the two groups. Dronedarone reduced the incidence of hospitalisation due to cardiovascular events or death [23].

In our study, dronedarone was well-tolerated, and the reported adverse drug reactions were in line with current knowledge. The most frequently reported reason for drug withdrawal was recurrence of AF and therefore not related to safety per se. With respect to laboratory values, the rate of abnormal liver function tests was substantially higher in IMPULS (10.9 %) compared with ATHENA (0.5 %) which might be due to the fact that in the latter trial there were no scheduled hepatic tests. Conversely, in ATHENA an increase in serum creatinine was noted in 4.7 %, compared with 0 % in our study. A recent retrospective database analysis on all 4856 patients treated with dronedarone in Sweden during 2010–2012, i.e., before the implementation of restrictions in the labelling of the drug, showed that patients selected for treatment were low-risk and had lower mortality than expected from the general population, or than AF patients on other antiarrhythmic medication [16]. Further, the risk of incident liver disease was significantly lower among dronedarone patients than among other AF patients (HR 0.57; 95 % CI 0.34–0.92) [16].

It is important to represent the patient perspective in the management of AF [24]. Thus, the number of studies that reported QoL in AF has steadily increased in the last years. The instrument ‘typically used’ is the SF-36, but the SF-12 has also been shown to provide robust results in the Birmingham Atrial Fibrillation Treatment of the Aged study [25]. In IMPULS, two questionnaires were administered in a complimentary manner to assess QoL, namely the SF-12 and EQ-5D VAS as generic and the AF-QoL as disease-specific instrument [15, 16]. Generic instruments document general aspects of physical, mental or social functionality, which can similarly be compromised in diverse diseases, and can be compared across these diseases using the instruments. However, they are often less sensitive [19, 20] as health improvement and QoL instruments are often not represented by a generic tool sufficiently [26]. Disease-specific questionnaires such as the AF-QoL focus on typical aspects of the disease, which may be experienced subjectively very differently by patients.

As it has been performed in a similar setting (office-based physicians in Germany), the MOVE cross-sectional study is particularly useful to compare results [27]. On the 100-point VAS, the 3354 patients overall had a value of 68 ± 18 points (paroxysmal 70, persistent 68, permanent 66 points) and thus a lower value compared with IMPULS (66 at baseline, 74 at FU). As in MOVE, [27] QoL scores were slightly worse in women compared to men, for all types of AF. It was notable that during the FU of the study in IMPULS, QoL on all instruments was substantially improved with the greatest effect already at the first FU visit. The observed improvement of 11 points on the VAS corresponds to a clinical improvement of about one category in the European Heart Rhythm Association (EHRA) score (which is reported in four classes) and thus represents a significant effect [28]. This finding may be due to the clinical effect of dronedarone, but could also be an unspecific consequence of intensive care of patients in the context of this study.

### Limitations

A number of limitations need to be addressed when the current results are interpreted. Given IMPULS was an open-label non-randomised study, different biases can obscure any true causal association [29]. As participating centres may have more scientific interest in particular in QoL investigations, results may not reflect outcomes in less well-organized institutions. Clinical decisions of the treating physicians may assign selected patients to drug treatment guided by disease severity, presence of comorbidities and other factors. This can potentially introduce allocation or channelling bias and confound the association between treatment and outcomes. The sample size was relatively small, and therefore the study was not powered to detect previously unknown, rare side effects of dronedarone. FU periods longer than 1 year are desirable to assess the long-term effects of the drug.

In conclusion, in this contemporary study on the use of dronedarone under clinical practice various dimensions of QoL of patients with AF were improved in the long term. No previously unknown safety issues were identified.
